# COVID-19 and Dental Education: the Experience of the Dental School from the University of Milan

**DOI:** 10.1007/s40670-022-01531-y

**Published:** 2022-03-17

**Authors:** Elena Maria Varoni, Andrea Sardella, Giovanni Lodi, Marcello Iriti, Antonio Carrassi

**Affiliations:** 1 Dipartimento Di Scienze Biomediche, Chirurgiche Ed Odontoiatriche, University of Milan, Via Beldiletto 1/3, 20142 Milan, Italy; 2 Dipartimento Di Scienze Agrarie E Ambientali, University of Milan, Milan, Italy

**Keywords:** Distance education, Coronavirus, Education technology, e-Learning, Online learning, Pandemic

## Abstract

In late February 2020, Lombardy became one of the outbreak areas of the novel coronavirus pandemic, leading to the revolution of traditional teaching. Here, we describe our teaching experience at the Dental School of the University of Milan, with a focus on the management of distance learning and clinical training. Distance education was enhanced with streaming lectures and videos of clinical procedures. Students’ opinions on this “digital revolution” were very positive, despite few technical and organizational problems. For assuring the clinical training, we completely renovated the structural architecture from open spaces to closed spaces. The pandemic changed dental education with future repercussions.

The coronavirus disease 2019 (COVID-19) pandemic is expected to have significantly affected the education of future healthcare professionals [1]. Medical education and healthcare systems have been strongly perturbed due to the need to deliver medical training and lectures safely despite the health emergency in progress.

Thanks to the recent advances in technologies and social media, distance learning has been regarded as the most suitable solution to maintain learning processes in exceptional and emergency situations, including COVID-19 pandemic. All around the world, thus, medical schools changed the way of teaching by organizing new distance learning platforms for contents and remote delivery of lectures, the use of question banks, panel discussion, and online interactive resources [2–5], with variable satisfaction rates among students.

From March 2020 to present, the Italian universities experienced, due to the COVID-19 pandemic, the suspension of traditional face-to-face teaching activities. Soon after the first outbreak, the distance teaching was immediately started. Later, when the infections began to decline, the so-called mixed modality teaching was introduced, where only a small number of booked students, not more than 50% of the members, could participate in a face-to-face lecture, while the others followed simultaneously on a remote telematics platform.

In this short communication, we have described our teaching experience at the Dental School of the University of Milan, with a focus on the management of distance learning and clinical training. Literature, indeed, is scanty in providing information on how to face COVID-19 pandemic in dental schools, and only few studies that share the approach applied to tackle the pandemic in the field of academic teaching are available [6, 7]. Most of them, in Europe [8], the USA [1], Canada [9], and India [10], were closed during the waves of pandemic. In addition face-to-face teaching was replaced with online learning. Clinical teaching was completely suspended and, for months, dental students had no possibility of face-to-face contact with tutors and patients.

**Table 1:** Safety procedures during clinical activities as implemented according to the international guidelines available for prevention and protection against SARS-CoV-2 [7, 14–16]

Post-COVID-19 safety recommendations for patient management	Pre-COVID-19 routine protocols for patient management
Telephone triage carried out the day before the procedure scheduled; Temperature control, masks, and hand hygiene for all subjects before entering the clinic; Distancing and mandatory use of masks for patients in waiting rooms; Additional triage conducted by nurses for all patients before the patient enters the operational clinical areas; Use of individual protective equipment for healthcare workers: caps, visors, FFP2, gloves, water-repellent gowns, footwear; Hand hygiene before and after a single procedure and before and after wearing disposable gloves; Rinsing the patient’s mouth with two mouthwashes before all the procedures (including visits), 1% hydrogen peroxide and 0.2% chlorhexidine; To favor the use, whenever possible, for the first aid of drug therapy, reducing the need of procedures that produce aerosol; Management of the patient with procedures that provide for no or minimal aerosol production and that are conducted with four hands, in case of aereosol; Disinfection and ventilation methods at the end of each single treatment; Identification of separate routes for incoming and outgoing patients	No telephone triage needed; No temperature control, masks, and hand hygiene for all subjects before entering the clinic; No distancing and mandatory use of masks for patients in waiting rooms; Triage conducted by nurses for all patients before the patient enters the operational clinical areas; Use of individual protective equipment for healthcare workers: caps, visors, surgical mask, gloves; Hand hygiene before and after a single procedure and before and after wearing disposable gloves; No need of pre-procedural rinsing for all the procedures; Management of the patient with the appropriate procedures, regardless aerosol production; No need to preferentially use drug therapy for first aid urgency and no need to limit aerosol-generating procedures; Management of the patient with appropriate procedures regardless aerosol production; Disinfection methods at the end of each single treatment, without need of ventilation; No need of separate routes for incoming and outgoing patients

To get a clearer picture in Europe, the Association for Dental Education in Europe (ADEE) distributed two questionnaires to the dental schools in Europe: the first one at the end of March 2020 and the second one at the beginning of June 2020. The first questionnaire [11] highlighted that half of clinics were completely closed, while the other half were operative just for urgencies and accessible only to the academic and hospital staff. Face-to-face teaching and academic exams were suspended and replaced by online lectures. Most of the respondents reported that the COVID-19 pandemic will change the future of dental education. The ADEE second questionnaire mainly focused on what could be the consequences of the pandemic on the architecture of dental schools and on the costs that dental schools would have to face, in order to promote the maximum operational safety of students, patients, and staff [12]. Over 60% of the respondent stated that there would be a need for major architectural changes and complete renovation, changing from open-space structures to closed rooms, the latter being more effective to contain aerosol. Most dental schools foresaw an increase in the costs for the personal safety protection devices and for the structural modifications, besides an increase in the time needed for performing a single procedure.

Italian dental schools had to face these same organizational challenges. In Italy, the fourth and fifth years of the dental curriculum require that the students should be progressively exposed to simulated and clinical activities with a gradual increase in their operational involvement. The sixth year is almost completely dedicated to clinical activities that students perform directly on patients with the support of a tutor. The spaces and patients where students work are derived from the National Health System. The Dental Clinic at the San Paolo Hospital has 43 dental units, which are involved in providing care through the National Health System, including dental urgencies.

As in most Italian and European dental schools, the impact of the COVID-19 pandemic on the clinical and educational activities has been dramatic.

In March 4, Italy was placed in lockdown. On the same day, the Permanent Conference of Italian Rectors suspended face-to-face teaching activities throughout the country, inviting all universities to use distance learning for theoretical lessons and suspend clinical training, while maintaining just research activities [13]. In March 9, in Lombardy, all the hospitals suspended outpatient therapies, with the exception of the activities related to the emergency/urgency departments or to those conditions that could not be postponed. Dental treatments could be provided exclusively by the academic, resident, and hospital staff.

Immediately, our clinic adopted the international safety clinical guidelines to contain SARS-CoV-2 diffusion [7, 14–16]. The guidelines were easily followed (Table 1), except during the first pandemic weeks, in which not all personal protective equipment (PPE) were always available, in particular, daily supply of water-repellent single-use gowns.

The main problem was the spatial architecture of the clinical units. Our dental clinic, like most European dental schools, is basically structured as a large open space due to the need for supervising professors to control the procedures and to move among students, tutors, and patients (Fig. 1). Of the 43 available units, only 10 were placed in closed rooms. During the pandemic months, we were forced to reduce of about 75% the aerosol-generating procedures, reserving them just for patients who could not be postponed, such as patients with acute pain/infection, patients receiving hematopoietic/organ transplant, or patients undergoing radio- or chemotherapy. A similar issue occurred in the waiting room due to safety distances that created a space, as tent structure, placed in the entrance courtyard of the dental clinic (Fig. 2).

**Fig. 1:**
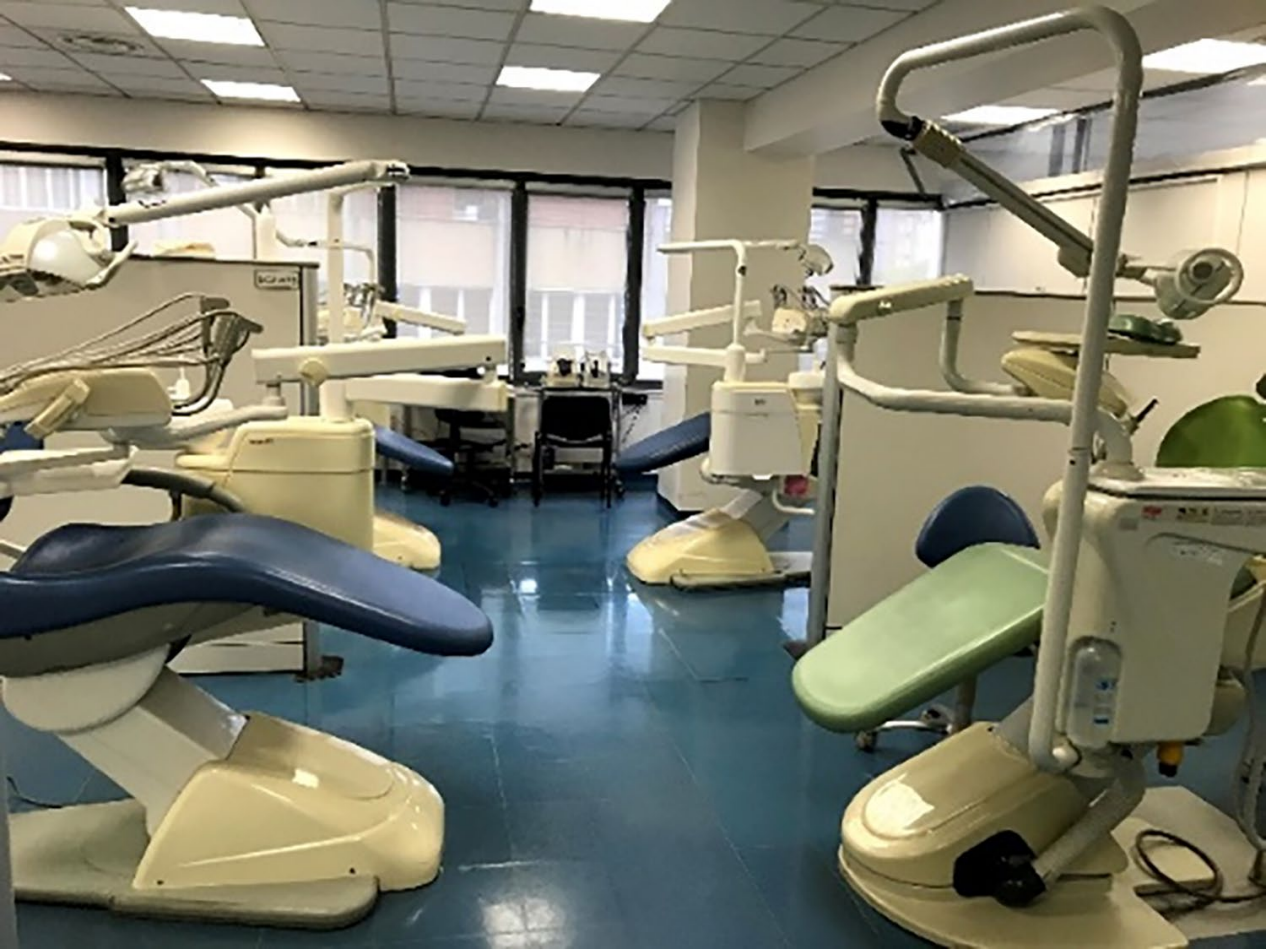
Typical arrangement of dental units in our dental school previous to COVID-19 pandemic: open space for dental chairs

**Fig. 2:**
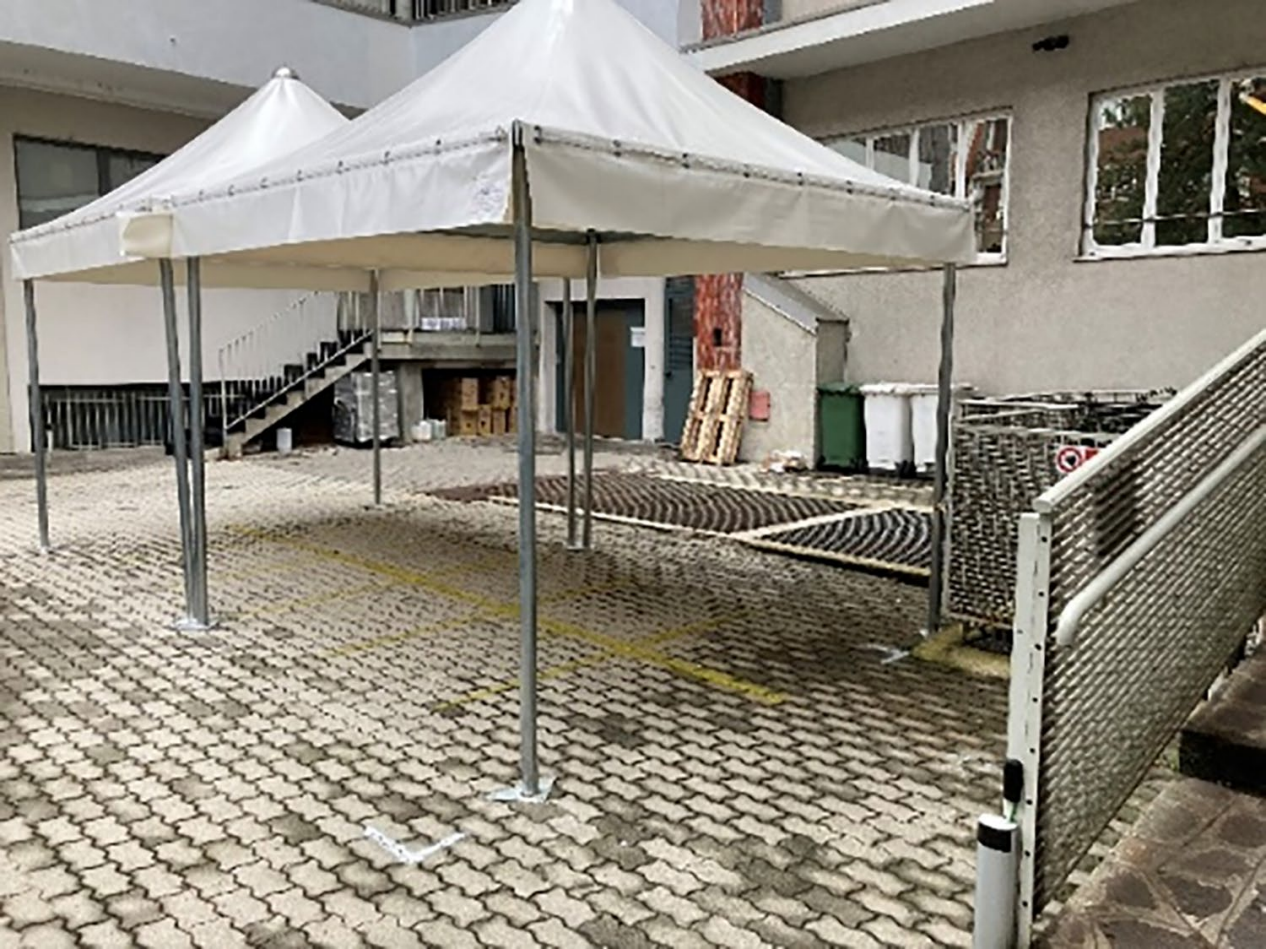
An open waiting room created in the courtyard of the Dental Clinic, in order to keep the correct distance among waiting patients

Figure 3A, B show the healthcare activity, in terms of total number of procedures delivered each month, during the period from January to November 2020 as compared with 2019. A significant reduction of routine procedures occurred, passing from 9877 procedures from February to November 2019 to 6657 procedures in the same period of 2020. Less dramatic was the decrease of urgencies that decreased from 4873 in February–November 2019 to 4210 in February–November 2020. Interestingly, an increase in dental emergencies could be recorded in June, July, and August 2020 as compared to that in 2019, and it is probably ascribed to the end of the pandemic lockdown, which was lifted in May 2020

**Fig. 3:**
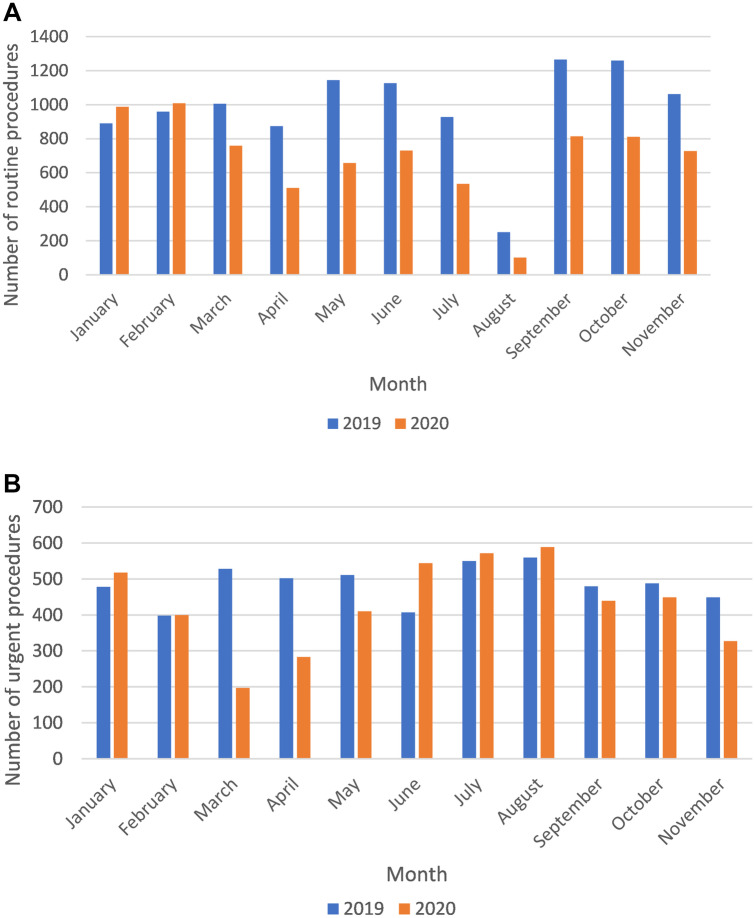
**A** Number of outpatients visited during the period January–November 2019 and 2020. **B** Number of urgent treatments during the period January–November 2019 and 2020

Overall, students lost a significant part of their clinical training, causing damage that will be difficult to remedy. Videos of clinical procedures were utilized, but, without hands-on practice, their clinical competence has certainly suffered. In a survey conducted on dental students, about 10% of respondents declared to be anxious about the future of dentistry, and, in some cases, they reported to think to change the career post-graduation plans [17].

The training facility was completely renovated with closed dental units (Fig. 4) to adhere to regulations regarding COVID-19. At the end of this restructuring, the facilities went from 43 to 32 units; thus, clinical activity will be limited even after the pandemic. We can eventually deliver dental treatments to less patients than pre-COVID-19 period, and dental students have fewer opportunities to learn. The lack of an “open-space approach” limits the possibility of immediate supervision by tutors and professors, suggesting the need to rethink the “student to supervisor” numerical ratio.

**Fig. 4:**
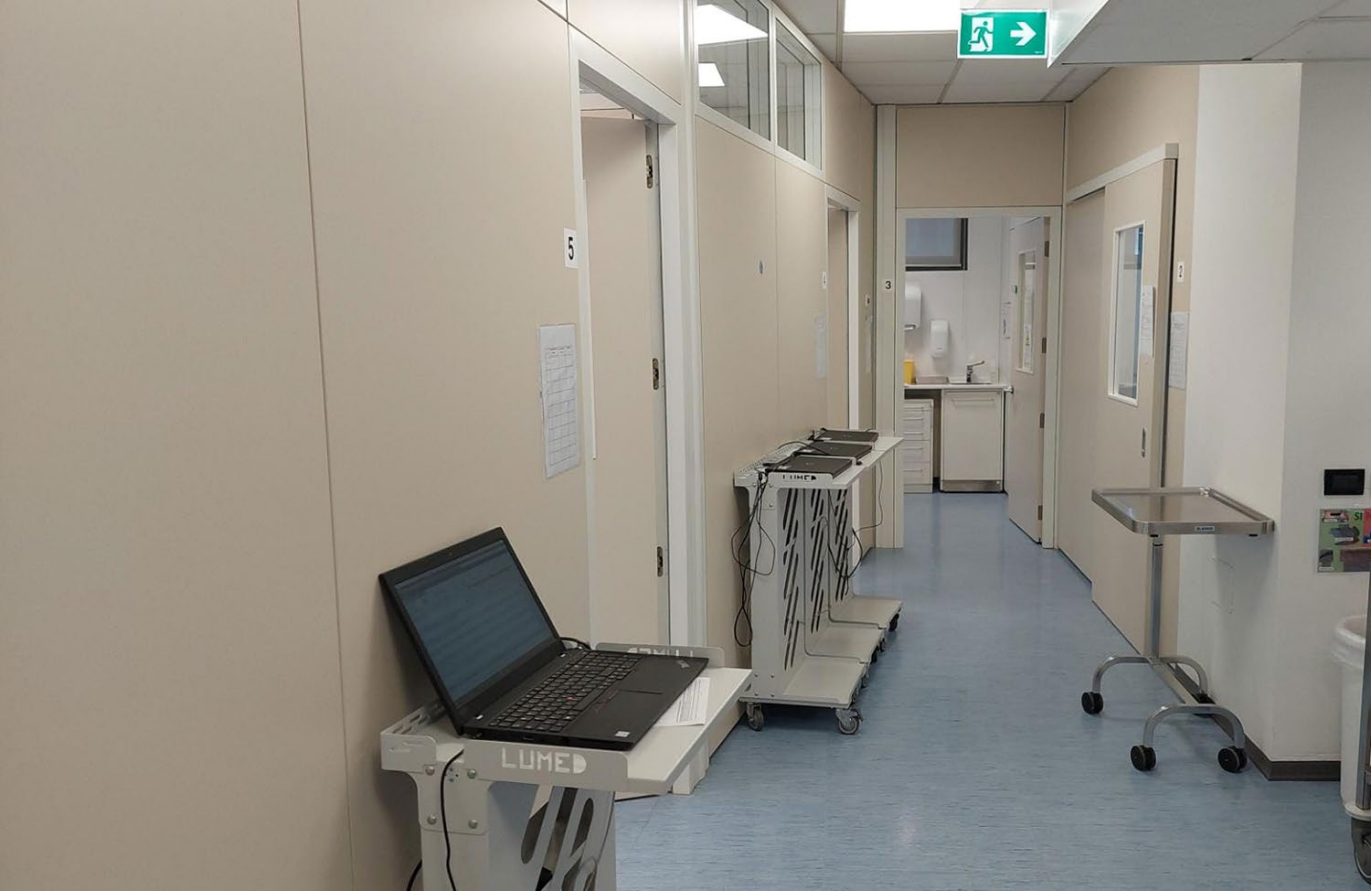
Arrangement of dental units in our Dental School after the reconstruction due to COVID-19 pandemic: closed work space for dental chairs

About theoretical lessons, thanks to the commitment and strong support of our university administration, it was possible to replace face-to-face teaching with online learning in a synchronous mode very quickly. In May 2020, the University of Milan Press published the data of a survey that aimed to investigate the students’ opinions on the “digital revolution” in teaching, which suddenly took off in the COVID-19 emergency. The aim was to understand how the students from all the academic courses perceived the online teaching. A questionnaire was distributed to 17,387 students of the University of Milan (not exclusively dental students), about 70% of those who usually attend classes in the second semester. Over 85% of the students expressed a positive opinion on distance learning [18]. However, students significantly acknowledged the disadvantage as the loss of direct contact with their classmates and professors.

The University of Turin and the “Italian Center for Research on Universities and Higher Education System” published a report entitled “Italian academics and distance learning during the COVID-19 emergency” [19]: 3812 professors and researchers from Italian universities were asked to complete a questionnaire on this topic. About 72% of respondents succeeded in activating remote teaching by 13 March; 75% were satisfied with the distance learning experience; 80% managed to complete their didactic program, with a prevalent use of live streaming lectures. These results are surprising if we consider that only 9% of the teachers interviewed previously had a distance teaching experience. In addition, during the first wave, professors had very little time to transform their traditional course into an online course, which takes many hours of work, and few had already received institutional training on online teaching technologies. In our university, several further problems emerged in the first months of distance teaching, including malfunctions of computers; connections with frequent loss of signal; and risks for privacy, data, video, and image protection.

In conclusion, the short-term effects of the COVID-19 pandemic on our dental school have been extremely negative. In the long term, we are being forced to develop a future vision for our activities, in the perspective of optimally containing the transmission of COVID-19. We decided, indeed, to renovate the clinics, with close dental units, removing the open-space architecture and not installing high-volume evacuation (HVE). Other schools have probably taken different paths. The sharing of different teaching experiences, as we did in this commentary, is pivotal to compare different academic realities and to implement teaching and clinical training in a safe and effective way during pandemics.
